# Cardiac-targeting magnetic lipoplex delivery of SH-IGF1R plasmid attenuate norepinephrine-induced cardiac hypertrophy in murine heart

**DOI:** 10.1042/BSR20130107

**Published:** 2014-10-02

**Authors:** Yiping Xu, Xuebiao Li, Minjian Kong, Daming Jiang, Aiqiang Dong, Zhonghua Shen, Qunjun Duan

**Affiliations:** *Children’ Hospital Zhejiang University School of Medicine Hangzhou 310003, China; †Cardiothoracic Surgery, Department of Second Affiliated Hospital, School of Medicine, Zhejiang University, Number 88, Jie Fang Road, Hang Zhou, Zhejiang Province 310009, China

**Keywords:** cardiac hypertrophy, gene therapy, IGF1R, magnetofection, AKT, protein kinase B, ANP, atrial natriuretic peptide, AUC, area under the curve, β-MHC, β-myosin heavy chain, ERK, extracellular-signal-regulated kinase, HW:BW, heart weight-to-body weight, IGF-1R, type 1 insulin-like growth factor receptor, LVID;d, left ventricular diastolic dimension, LVID;s, left ventricular internal diameter at end-systole, LVPWT, left ventricular posterior wall thickness, EF%, ejection fraction, %FS, per cent fractional shortening, PI3K, phosphoinositide 3-kinase, shRNA, small-hairpin RNA, SPION, superparamagnetic iron oxide nanoparticle

## Abstract

Recent studies have demonstrated a number of molecular mechanisms contributing to the initiation of cardiac hypertrophy response to pressure overload. IGF1R (insulin-like growth factor-1 receptor), an important oncogene, is overexpressed in hypertrophic heart and mediates the hypertrophic pathology process. In this study, we applied with liposomal magnetofection that potentiated gene transfection by applying an external magnetic field to enhance its transfection efficiency. Liposomal magnetofection provided high efficiency in transgene expression *in vivo*. *In vivo*, IGF1R-specific-shRNA (small-hairpin RNA) by magnetofection inhibited IGF1R protein expression by 72.2±6.8, 80.7±9.6 and 84.5±5.6%, at 24, 48 and 72 h, respectively, after pGFPshIGF1R injection, indicating that liposomal magnetofection is a promising method that allows the targeting of gene therapy for heart failure. Furthermore, we found that the treated animals (liposomal magnetofection with shIGF1R) showed reduced septal and posterior wall thickness, reduced HW:BWs (heart weight-to-body weights) compared with controls. Moreover, we also found that liposomal magnetofection-based shIGF1R transfection decreased the expression level of p-ERK (phosphorylated extracellular-signal-regulated kinase)1/2, p-AKT1 (phosphorylated protein kinase B1) compared with untreated hearts. These results suggested that liposomal magnetofection-mediated IGF1R-specific-shRNA may be a promising method, and suppression the IGF1R expression inhibited norepinephrine-induced cardiac hypertrophic process via inhibiting PI3K (phosphoinositide 3-kinase)/AKT pathway.

## INTRODUCTION

Recent studies have demonstrated a number of risk factors for heart failure, and the molecular mechanisms that contribute to the initiation of heart failure are incompletely understood [1,2]. These results suggest that heart failure, the leading contributor of human morbidity and mortality in the developed world, could be prevented or reverted, at least in experimental models [3–5]

IGF1R (insulin-like growth factor-1 receptor), an important tyrosine kinase receptor involved in cell metabolism, proliferation, differentiation, apoptosis, chemoresistance and angiogenesis, plays an important role in carcinogenesis. Moreover, the activation of IGF1R expressions related with malignant transformation, poor prognosis [6–10]. Recent studies have shown mammalian ageing and ageing-associated diseases are associated with dysregulation of IGF–Akt (protein kinase B) signalling. In the heart, short-term activation of IGF-Akt signalling pathway promotes physiologic growth, whereas sustained hyper-activation leads to the development of pathologic hypertrophy and heart failure [11–13].

Gene therapy is a promising method for human diseases. However, no effective and safety enough vector was developed, which limited its clinical use. Viral vector systems have been developed to transfer gene into cells with high efficiency, but some disadvantages such as limitations on plasmid insert size and safety issues, including mutagenic effects and induction of inflammatory responses were also concerned. Non-viral vectors were also designed as alternation of viral vectors. Actually, the non-viral vectors do not carry shRNA (small-hairpin RNA) or plasmid DNA to cells efficiently *in vitro* and even lower efficiency *in vivo*, whereby targeting delivery of therapeutic genes *in vivo* is required. Magnetofection, a novel and efficient gene delivery method, in which nucleic acids are conjuncted with magnetic nanoparticles in combination with cationic lipid, shows great promise in addressing the limitations discussed [14–22]. As in our previous work, magnetic lipoplex delivery of short hairpin RNA suppressed IGF1R overexpression of lung adenocarcinoma A549 cells *in vitro* and *in vivo* [23]. Magnetofection, exploiting the magnetic force exerted upon gene vectors associated with magnetic particles enhanced gene transfection not only into cultured cells *ex vivo*, but *in vivo* gene delivery into targeted tissues and organs in the body under the control of a magnetic field [24–26].

Although, the potential of using SPIONs (superparamagnetic iron oxide nanoparticles) to mediate gene delivery has been elegantly demonstrated in various cell lines and primary human cells, very few studies have ever been systematically assessed the feasibility of liposomal magnetofection for gene transfer to heart *in vivo*.

We addressed this issue by applying plasmid DNA conjugated with SPIONs in combination with Lipofectamine 2000 (Lip2000, Invitrogen) to transfer to heart *in vivo*, and thus evaluating the effects of shIGF1R on cardiac hypertrophic process.

## MATERIAL AND METHODS

### Cardiac hypertrophy disease model in murine heart

All experiments were performed according to protocols approved by the guideline of Zhejiang University's Institutional guidelines established for animal care and use. All animals were obtained from Zhejiang Academy of Medical Science (Hangzhou, China) and maintained in a clean environment. Eight-week-old male C57BL/6 mice were used in this study. The mice received a continuous infusion of norepinephrine or vehicle (saline) at a dose of 15 mg/kg/day for 11 days.

### Echocardiography, morphometry and histology analysis

Transthoracic echocardiography was performed in awaked mice with VisualSonics Vevo® 2100 Imaging System. The echocardiographer was blinded to the experimental status of the mice. Cardiac hypertrophic parameters such as LVID;s (left ventricular internal diameter at end-systole) and LVID;d (left ventriular diastolic dimension) LVPWT (left ventricular posterior wall thickness), ISVT (interventricular septal thickness), ejection fraction (EF%), left ventricular shorting rate (FS%) were calculated to determine cardiac hypertrophy. Histological analysis was performed in paraffin-embedded heart sections. HW/BT (heart weight-to-body weight) was calculated to determine cardiac hypertrophy. Cardiac myocyte cross-sectional area was also calculated in high-power fields from the left ventricular region.

### Liposomal magnetofection-based gene therapy *in vivo*

Liposomal magnetofection-based gene therapy *in vivo* was performed as described [23]. The mice were randomly divided into two groups (*n*=8 per group): control group liposomal magnetofection group. A volume of 200 μl saline was injected into the mice via the tail vein, and in the liposomal magnetofection group, a complex of pGFPshIGF1R (50 μg/mouse):combiMAG (50 μg/mouse):Lip2000 (125 μl/mouse) was injected into the mice via the tail vein when the mouse chest exposed upon a 400 mT magnetic field. In the liposomal magnetofection group, Nd–Fe–B magnet (400 mT) was held onto the thoracic surface for 20 min following the injection. The magnetic field was then removed. At 24 h post-injection, the mice in the control and liposomal magnetofection group were killed by cervical dislocation and the hearts were quickly removed and analysed as a snap-frozen section (5 μm) using a freezing microtome (Leica CM1900). The GFP (green fluorescent protein) expressing cells in the heart tissue sections were visualized using a fluorescence microscope.

### Western blot analysis

At 24, 48, 72 h post-injection, mice in the control group and treatment group were killed and the hearts were quickly removed. Protein was extracted from these heart tissues and Western blot analysis was performed as previously described [23]. Briefly, 60 μg of the total tissue proteins from heart tissues of mice (30 μg for β-actin) were separated on 10% SDS–PAGEs, and then analysed using anti-IGF-1R, anti-AKT, anti-p-akt, anti-ERK (extracellular-signal-regulated kinase)1/2, anti-p-ERK1/2 (Santa Cruz).

### Quantitative real-time PCR

The mRNA levels of β-MHC (β-myosin heavy chain), ANP (atrial natriuretic peptide) were determined by real time-PCR using a QuantiTect SYBR Green real-time PCR. Kit (TAKARA). Total RNA was isolated from samples with Trizol reagent (Life tech) according to the manufacturer's instructions. Primers were designed to generate short amplification products. The sequences of the specific primers were: IGF1R, 5′-ACCGCAACTACCGCTTCCCC-3′ (forward) and 5′-CCGCG GATGACCGTGAGGTT-3′ (reverse); β-MHC, 5′-CAGACATAGAGACCTACCTTC-3′ (forward) and 5′-CAGCATGTCTAGAAGCTCAGG-3′ (reverse); ANP, 5′-CTCCGATAGATCTGCCCTCTTGAA-3′ (forward) and 5′-GGTACCGGAAGCTGTTGCAGCCTA-3′ (reverse); β-actin, 5′-TGTGTCCGTCGTGGATCTGA-3′ (forward) and 5′-CCTGCTTCACCACCTTCTTGA-3′ (reverse). Real-time PCR was performed in 20 μl reaction volumes using 10 pmol of primers. Reverse transcription was performed at 37°C for 15 min, and cDNA was amplified for 40 cycles: 95°C for 5 min, 95°C for 15 s, 60°C for 34 s, and 95°C for 15 s. β-actin was used as an internal control to calculate the relative abundance of mRNAs. The relative expression between treatments was calculated using the following equation: relative gene expression=2^−(ΔCtsample−ΔCt control)^.

### Statistical analyses

Data were presented as mean ± S.D. from at least three parallel experiments. One-way ANOVA and Student's *t* test were utilized and *P*<0.05 was considered statistically significant.

## RESULTS

### Liposomal magnetofection allowed site-specific gene delivering *in vivo*

**Figure 1:**
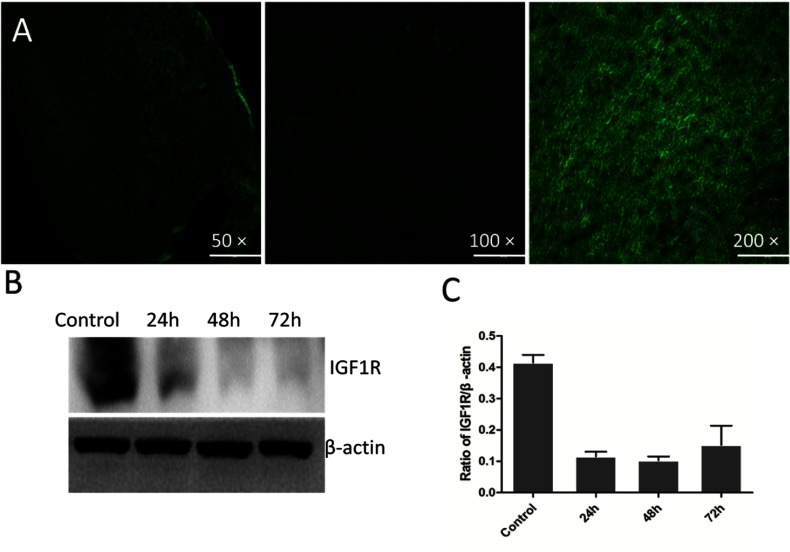
Efficiency of magnetofection-based transfection and expression of IGF-1R (**A**) High efficiency of liposomal magnetofection-based gene transfection *in vivo.* Magnification=50×, 100×, 200×, respectively. (**B**) Down-regulation of IGF-1R expression in mouse heart by delivered shRNA specific for IGF-1R using liposomal magnetofection *in vivo*. (**C**) shRNA transfection by liposomal magnetofection significantly inhibited IGF-1R protein expression at 24, 48 and 72 h, *P<0.01 versus control (saline injection). Bars denote standard errors. *N*=3.

To evaluated the feasible target-specific gene therapy *in vivo* with liposomal magnetofection, pGFPshIGF-1R: combiMAG:Lip2000 complex was injected via the tail vein in the direction of the magnetic field. High GFP expression was observed in the heart tissue suggesting that liposomal magnetofection could be a specific method to promote uptake of plasmid DNA into specific target sites (Figure 1A). Western blotting showed high interference efficiency of shRNAs delivered by liposomal magnetofection in heart at 24, 48 and 72 h. The results showed that the silencing efficiency of shRNAs delivered by liposomal magnetofection reached 72.2±6.8, 80.7±9.6 and 84.5±5.6%, at 24, 48 and 72 h, respectively (Figures 1B and 1C). These results supported that liposomal magnetofection was an efficient way, which allowed target gene therapy.

### Norepinephrine induced cardiac hypertrophy and overexpression of IGF1R

**Figure 2:**
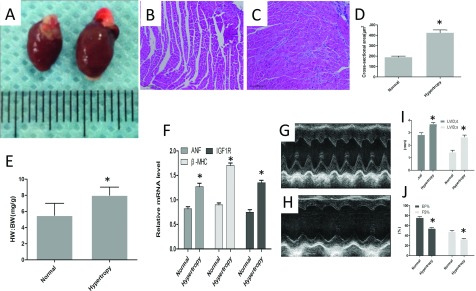
Cardiac dysfunction in norepinephrine administrated mice (**A**) Morphological characteristics of isolated heart from control group and norepinephrine administrated group. Histological evaluation the cross-sectional area of heart tissue from normal group (**B**), norepinephrine administrated mice (**C**), Magnification=200×. (**D**) Norepinephrine administration increased the cross-sectional area compared with control, *P<0.05. (**E**) Ratio of HW/BW within two group,*P<0.05. (**F**) The mRNA level of IGF1R, β-MHC, ANP were up-regulated in norepinephrine administrated heart tissues, *P<0.05 versus control. Echocardiographic evaluation of heart function in normal mice (**G**) and hypertrophic heart (**H**). (**I**) Norepinephrine administration decreased the fractional shortening of left ventricle (FS%) and EF%,*P<0.05 versus control. (**J**) Norepinephrine administration induced cardiac hypertrophy. Parameters of cardiac hypertrophy were evaluated, *P<0.05 versus control. LVDd;d, diastolic left ventricle internal dimension; LVDd;s, left ventricular diastolic dimension

Pressure overload mediated ventricular myocardium hypertrophic decreased myocardial wall stress, activated maladaptive hypertrophic pathways, leading to impaired systolic function, cardiac myocyte apoptosis and fibrosis (Figure 2). Histological analysis showed increased cardiomyocyte cross-sectional area and HW (heart weight)/BW (body weight) in norepinephrine-treated mice compared with control mice. At 11 days after norepinephrine using, echocardiographic analysis was performed to calculate the key hypertrophic parameters, such as LVID;s and LVID;d, LVPWT, ISVT, EF% (ejection fraction), left ventricular shorting rate (FS%) to determine cardiac hypertrophy. Echocardiography revealed increased septal and posterior wall thickness, HW:BWs (heart weight-to-body weights) compared with controls. Importantly, norepinephrine administration *in vivo* increased end-diastolic volumes. At 11days, mice administrated with norepinephrine exhibited a significant decrease in cardiac EF% and depressed %FS (per cent fractional shortening); and the expression of IGF1R was up-regulated after norepinephrine administration. In addition, the mRNA (messenger RNA) expression level of ANF and β-MHC, markers of left ventricular hypertrophy, were also significantly higher. Histological analysis showed the increased cardiomyocyte cross-sectional area in norepinephrine-treated mice, compared with control mice.

### Liposomal magnetofection-mediated site-specific gene delivering attenuated cardiac hypertrophy *in vivo*

Liposomal magnetofection mixture was continually administered at the first 4-days via tail vein injection combined norepinephrine administration. At 11 days, the baseline of cardiac function was evaluated. ShIGF1R-treated mice exhibit little echocardiographic, gross anatomical changes because of cardiac IGF1R down-regulation. This indicated that long-term silencing of IGF1R had no significant effect on myocardial function in the absence of pathological stresses.

**Figure 3:**
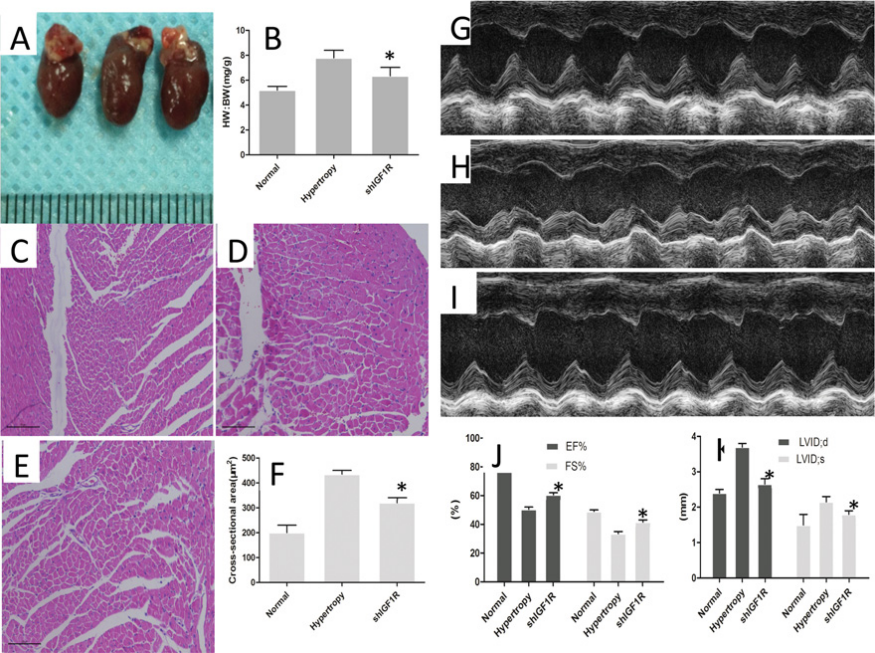
Effects of shIGF1R administration on cardiac hypertrophic process *in vivo* (**A**) Morphological characteristics of isolated heart from different groups. (**B**) Ratio of HW/BW in different group,*P<0.05. Histological evaluation the cross-sectional area of heart tissue from normal group (**C**), shIGF1R administrated group (**D**), saline-treated group (**E**), Magnification=200×. (**F**) shIGF1R administration attenuated the cross-sectional area compared with control, *P<0.05. Echocardiographic evaluation of heart function in normal mice (**G**), hypertrophy (**H**), shIGF1R-treated mice (**I**). (**J**) shIGF1R administration increased the fractional shortening of left ventricle (FS%) and EF%,*P<0.05 versus control. (**K**) Parameters of cardiac hypertrophy:LVDd;d, diastolic left ventricle internal dimension; LVDd;s were evaluated, *P<0.05 versus control.

At 11 days post-norepinephrine administration, mice were sacrificed and heart tissues were collected. Echocardiographic results showed that silencing IGF1R attenuated hypertrophic remodelling as compared with controls. Specifically, liposomal magnetofection-shIGF1R mixture-treated mice showed the reduced septal and posterior wall thickness, as revealed in the echocardiograms, and had reduced HW:BWs compared with no shIGF1R-treated controls. Importantly, liposomal magnetofection-shIGF1R transfection *in vivo* reduced end-diastolic volumes with elevated ventricular pressures indicating that silencing IGF1R expression protect against ventricular remodelling in the setting of pressure overload. At 11 days, mice treated with shIGF1R exhibited a increase in cardiac EF%, and significant increase in function with depressed %FS. Echocardiography revealed maintenance of ventricular volumes in mice treated with shIGF1R. shIGF1R treatment protected the myocardium from systolic failure. Histological analysis showed the decreased cardiomyocyte cross-sectional area in shIGF1R-treated mice (Figure 3).

### Treatment with sh-IGF1R-attenuated cardiac hypertrophy by inhibiting the PI3K/AKT signal pathway

**Figure 4:**
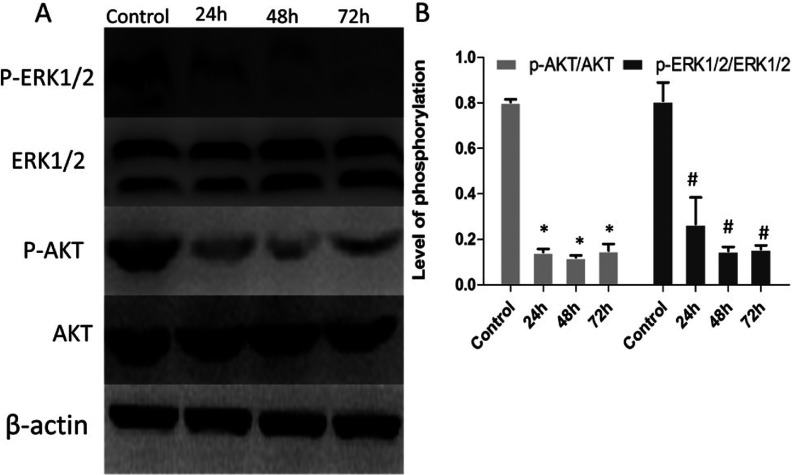
ShIGF1R treatment negatively regulated the IGF signalling pathway (**A**) Representative Western blots showed decreased expression of phosphorylation of IGF signalling-related proteins (ERK1/2, AKT) in shIGF1R-treated group compared with control. *N=* 6 per group. The ‘p-’ prefix indicates the phosphorylated form. (**B**) Data were presented as the mean ± S.D. For p-AKT, *P<0.001 versus normal heart tissues and for p-ERK1/2,#*P*<0.01 compared with normal heart tissues.

Various factors such as IGF-1, angiotensin II, endothelin, lead to the development of cardiac hypertrophy and that this requires a fully functional IGF-1 receptor. Studies also showed that the ERK (extracellular signal-regulated kinase) 1/2, Akt could be involved in downstream of IGF1/IGF-1R pathway [27]. The ERK can be activated by several hypertrophic stimuli such as fibroblast growth factors, ET-1 and K1-adrenergic agonists, TPA, stretching, and, bradykinin [28,29]. Activation of ERK exerted a common hypertrophic action. To elucidate whether IGF1R gene silencing by shIGF1R was associated with suppression of PI3K/AKT signal pathway, expression of these proteins in the heart tissues were assessed by Western blot. Protein samples were extracted from the heart tissues after post-injection day 1, day 2 and day 3. We noted that liposomal magnetofection-based shIGF1R transfection decreased the expression of p-ERK1/2, p-AKT1 compared with control hearts, suggesting that sh-IGF1R inhibited norepinephrine administration-induced cardiac hypertrophic process via inhibiting PI3K/AKT pathway (Figure 4).

## DISCUSSION

We successfully applied commercially available superparamagnetic iron-oxide nanoparticles in combination with Lipofectamine 2000-mediated gene delivery under the influence of an external static magnetic field in an NSCLC cell line (A549) *in vitro* and further achieved targeting transfection in nude mice.

We showed that magnetofection was a novel method of targeting therapeutic gene delivery for gene therapy of cancer [23].

Mice undergo a progressive left ventricle dilation followed a period of 11 days norepinephrine administration. Norepinephrine administration impaired the left ventricle remodelling inducing typical pattern of eccentric left ventricular hypertrophy, enlargement of the left ventricular chamber and an impaired wall thickening. Magnetic nanoparticles, iron oxide core composed a biocompatible polymeric coating, have been extensively studied as a method for localized drug delivery to tumours [30,31]. Surface properties of magnetic nanoparticles was associated with the transfection efficiency, especially the surface charge of the nanoparticles [32,33]. A positive surface charge has been shown several drug/gene delivery advantages. However, cationic nanoparticles suffer from a several drawback. First, cationic nanoparticles attract opsonizing proteins, which leads to rapid plasma clearance of the nanocarriers, such as short nanoparticle plasma half-life [34]. Secondly, performed magnetic targeting procedures involve the magnetic capture of nanoparticles administered locally to superficial tissues or subcutaneous tumours [35]. Following intravenous administration, magnetic nanoparticles have to be passively delivered to the tumour vasculature for subsequent magnetic capture. Thirdly, the amount of agent available for tumour uptake over time after systemic administration is known to be directly proportional to the area under its plasma concentration-time curve [AUC (area under the curve)] [36]. And surface modification with PEG (polyethylene glycol), termed PEGylation can increase the AUC [37]. In this study, we achieved highly efficiency of targeting transfection in mice applied with liposomal magnetofection. The expression of IGF1R in mouse heart was efficiently silenced.

Furthermore, we found that the absence of IGF1R caused a decrease left ventricular remodelling in conditions of sustained biomechanical stress, such as eccentric hypertrophy, thus accelerating the transition towards heart failure. We found that mice treated with shIGF1R exhibited a increase in cardiac EF%, significant increase in function with depressed %FS. Echocardiography revealed maintenance of ventricular volumes protecting the myocardium from systolic failure in mice treated shIGF1R.

IGFIR is related to the development of myocyte hypertrophy [38]. IGF1 increased protein synthesis in cardiac myocytes via activation of the ERK1/2 cascade pathway, and inhibitors of signal transduction on IGF1 can protect against cardiac myocyte hypertrophy [39]. The MAPKs (mitogen-activated protein kinases) cascade, such as ERK, c-JNK (c-Jun N-terminal kinase) and p38 are implicated as important regulators of cardiomyocyte hypertrophic growth.ERK1/2 signalling pathway activation stimulated a physiologic hypertrophy response associated with augmented cardiac function and partial resistance to apoptotsis [40].

The absence of IGF1R expression specifically reduced phosphorylation of ERK1/2, and substantially affect phosphorylation of AKT. The expression of p-ERK1/2, p-AKT in the heart tissues were significantly decreased compared with control hearts after sh-IGF1R post-injection day 1, day 2 and day 3. These results suggested that sh-IGF1R transfection inhibited norepinephrine administration-induced cardiac hypertrophic process via inhibiting PI3K/AKT pathway.

In conclusion, we successfully achieved targeting transfection to mice heart by applying commercially available superparamagnetic iron-oxide nanoparticles in combination with lipofectamine 2000-mediated gene delivery under the influence of an external static magnetic field, which provided a novel method of targeting therapeutic gene. Even more, silencing IGF1R expression maintained ventricular volumes protecting the myocardium from systolic failure.

## References

[1] Lloyd-Jones D., Adams R. J., Brown T. M., Carnethon M., Dai S., De Simone G., Ferguson T. B., Ford E., Furie K., Gillespie C. (2010). Heart disease and stroke statistics-2010 update: a report from the American Heart Association. Circulation.

[2] Donmez G., Guarente L. (2010). Aging and disease: connections to sirtuins. Aging Cell.

[3] Kahn A. J. (1972). Development, aging, and life duration: effects of nutrient restriction. Am. J. Clin. Nutr..

[4] Fontana L., Partridge L., Longo V. D. (2010). Extending healthy life span-from yeast to humans. Science.

[5] Weiss E. P., Fontana L. (2011). Caloric restriction: powerful protection for the aging heart and vasculature. Am. J. Physiol. Heart Circ. Physiol..

[6] Yin M., Guan X., Liao Z., Wei Q. (2009). Insulin-like growth factor-1 receptor-targeted therapy for non-small cell lung cancer: a mini review. Am. J. Transl. Res..

[7] Grothey A., Voigt W., Schober C., Muller T., Dempke W., Schmoll H. J. (1999). The role of insulin-like growth factor I and its receptor in cell growth, transformation, apoptosis, and chemoresistance in solid tumors. J. Cancer Res. Clin. Oncol..

[8] Spitz M. R., Barnett M. J., Goodman G. E., Thornquist M. D., Wu X., Pollak M. (2002). Serum insulin-like growth factor (IGF) and IGF-binding protein levels and risk of lung cancer: a case-control study nested in the beta-carotene and retinol efficacy trial cohort. Cancer Epidemiol. Biomarkers Prev..

[9] Yu H., Mistry J., Nicar M. J., Khosravi M. J., Diamandis A., van Doorn J., Juul A. (1999). Insulin-like growth factors (IGF-I, free IGF-I and IGF-II) and insulin-like growth factor binding proteins (IGFBP-2, IGFBP-3, IGFBP-6, and ALS) in blood circulation. J. Clin. Lab. Anal..

[10] Renehan A. G., Zwahlen M., Minder C., O’Dwyer S. T., Shalet S. M., Egger M. (2004). Insulin-like growth factor (IGF)-I, IGF binding protein-3, and cancer risk: systematic review and meta-regression analysis. Lancet.

[11] Holzenberger M., Dupont J., Ducos B., Leneuve P., Géloën A., Even P. C., Cervera P., Le Bouc Y. (2003). IGF-1 receptor regulates lifespan and resistance to oxidative stress in mice. Nature.

[12] Matsui T., Li L., Wu J. C., Cook S. A., Nagoshi T., Picard M. H., Liao R., Rosenzweig A. (2002). Phenotypic spectrum caused by transgenic overexpression of activated Akt in the heart. J. Biol. Chem..

[13] Matsui T., Nagoshi T., Rosenzweig A. (2003). Akt and PI 3-kinase signaling in cardiomyocyte hypertrophy and survival. Cell Cycle.

[14] Mykhaylyk O., Antequera Y. S., Vlaskou D., Plank C. (2007). Generation of magnetic nonviral gene transfer agents and magnetofection *in vitro*. Nat. Protoc..

[15] Plank C., Anton M., Rudolph C., Rosenecker J., Krotz F. (2003). Enhancing and targeting nucleic acid delivery by magnetic force. Expert Opin. Biol. Ther..

[16] McBain S. C., Yiu H. H., Dobson J. (2008). Magnetic nanoparticles for gene and drug delivery. Int. J. Nanomed..

[17] Plank C., Schillinger U., Scherer F., Bergemann C., Remy J. S., Krotz F., Anton M., Lausier J., Rosenecker J. (2003). The magnetofection method: using magnetic force to enhance gene delivery. Biol. Chem..

[18] Dobson J. (2006). Gene therapy progress and prospects: magnetic nanoparticle-based gene delivery. Gene Ther..

[19] Song H. P., Yang J. Y., Lo S. L., Wang Y., Fan W. M., Tang X. S., Xue J. M., Wang S. (2010). Gene transfer using self-assembled ternary complexes of cationic magnetic nanoparticles, plasmid DNA and cell-penetrating Tat peptide. Biomaterials.

[20] Gersting S. W., Schillinger U., Lausier J., Nicklaus P., Rudolph C., Plank C., Reinhardt D., Rosenecker J. (2004). Gene delivery to respiratory epithelial cells by magnetofection. J. Gene Med..

[21] Krotz F., de Wit C., Sohn H. Y., Zahler S., Gloe T., Pohl U., Plank C. (2003). Magnetofection-a highly efficient tool for antisense oligonucleotide delivery *in vitro* and *in vivo*. Mol. Ther..

[22] Schillinger U., Brill T., Rudolph C., Huth S., Gersting S., Krotz F., Hirschberger J., Bergemann C., Plank C. (2005). Advances in magnetofection-magnetically guided nucleic acid delivery. J. Magn. Magn. Mater..

[23] Chunmao W., Chao D., Minjian K., Aiqiang D., Jianfang Q., Daming J. (2011). Tumor-targeting magnetic lipoplex delivery of short hairpin RNA suppresses IGF-1R overexpression of lung adenocarcinoma A549 cells *in vitro* and *in vivo*. Biochem. Biophys. Res. Commun..

[24] Prosen L., Prijic S., Music B., Lavrencak J., Cemazar M., Sersa G. (2013). Magnetofection: a reproducible method for gene delivery to melanoma cells. Biomed. Res. Int..

[25] Prijic S., Scancar J., Romih R., Cemazar M., Bregar V. B., Znidarsic A., Sersa G. (2010). Increased cellular uptake of biocompatible superparamagnetic iron oxide nanoparticles into malignant cells by an external magnetic field. J. Membr. Biol..

[26] Prijic S., Prosen L., Cemazar M., Scancar J., Romih R., Lavrencak J., Bregar V. B., Coer A., Krzan M., Znidarsic A., Sersa G. (2012). Surface modified magnetic nanoparticles for immuno-gene therapy of murine mammary adenocarcinoma. Biomaterials.

[27] Haq S., Choukroun G., Lim H., Tymitz K. M., del Monte F., Gwathmey J., Grazette L., Michael A., Hajjar R., Force T., Molkentin J. D. (2001). Differential activation of signal transduction pathways in human hearts with hypertrophy versus advanced heart failure. Circulation.

[28] Bogoyevitch M. A., Glennon P. E., Andersson M. B., Clerk A., Lazou A., Marshall C. J., Parker P. J., Sugden P. H. (1994). Endothelin-1 and fibroblast growth factors stimulate the mitogen-activated protein kinase signaling cascade in cardiac myocytes. The potential role of the cascade in the integration of two signaling pathways leading to myocyte hypertrophy. J. Biol. Chem..

[29] Bogoyevitch M. A., Glennon P. E., Sugen P. H. (1993). Endothelin-1, phorbol esters and phenylephrine stimulate MAP kinase activities in ventricular cardiomyocytes. FEBS Lett..

[30] Dobson J. (2006). Magnetic nanoparticles for drug delivery. Drug Dev. Res..

[31] Alexiou C., Jurgons R., Schmid R. J., Bergemann C., Henke J., Erhardt W., Huenges E., Parak F. (2003). Magnetic drug targeting-biodistribution of the magnetic carrier and the chemotherapeutic agent mitoxantrone after locoregional cancer treatment. J. Drug Target..

[32] Mykhaylyk O., Vlaskou D., Tresilwised N., Pithayanukul P., Moller W., Plank C. (2007). Magnetic nanoparticle formulations for DNA and siRNA delivery. J. Magn. Magn. Mater..

[33] Koch A., Reynolds F., Kircher M., Merkle H., Weissleder R., Josephson L. (2003). Uptake and metabolism of a dual fluorochrome tat-nanoparticle in HeLa cells. Bioconjug. Chem..

[34] Weissleder R., Bogdanov A., Neuwelt E. A., Papisov M. (1995). Long-circulating ironoxides for MR-Imaging. Adv. Drug Deliv. Rev..

[35] Scherer F., Anton M., Schillinger U., Henke J., Bergemann C., Kruger A. (2002). Magnetofection: enhancing and targeting gene delivery by magnetic force *in vitro* and *in vivo*. Gene Ther..

[36] Blasberg R. G., Groothuis D. R. (1986). Chemotherapy of brain tumors: physiological and pharmacokinetic considerations. Semin. Oncol..

[37] Storm G., Belliot S., Daemen T., Lasic D. (1995). Surface modification of nanoparticles to oppose uptake by the mononuclear phagocyte system. Adv. Drug Deliv. Rev..

[38] Toyozaki T., Hiroe M., Hasumi M., Horie T., Hosoda S., Tsushima T., Sekiguchi M. (1993). Insulin-like growth factor I receptors in human cardiac myocytes and their relation to myocardial hypertrophy. Japan. Circ. J..

[39] Lavandero S., Foncea R., Pérez V., Sapag-Hagar M. (1998). Effect of inhibitors of signal transduction on IGF-1-induced protein synthesis associated with hypertrophy in cultured neonatal rat ventricular myocytes. FEBS Lett..

[40] Bueno O. F., De Windt L. J., Tymitz K. M., Witt S. A., Kimball T. R., Klevitsky R., Hewett T. E., Jones S. P., Lefer D. J., Peng C. F. (2000). The MEK1-ERK1/2 signaling pathway promotes compensated cardiac hypertrophy in transgenic mice. EMBO J..

